# Group B Streptococcal Infection and Activation of Human Astrocytes

**DOI:** 10.1371/journal.pone.0128431

**Published:** 2015-06-01

**Authors:** Terri D. Stoner, Thomas A. Weston, JoAnn Trejo, Kelly S. Doran

**Affiliations:** 1 Department of Biology, Center for Microbial Sciences, San Diego State University, San Diego, CA, United States of America; 2 Department of Pharmacology, School of Medicine, University of California San Diego, La Jolla, CA, United States of America; 3 Departmant of Pediatrics, School of Medicine, University of California San Diego, La Jolla, CA, United States of America; University of Kansas Medical Center, UNITED STATES

## Abstract

**Background:**

*Streptococcus agalactiae* (Group B *Streptococcus*, GBS) is the leading cause of life-threatening meningitis in human newborns in industrialized countries. Meningitis results from neonatal infection that occurs when GBS leaves the bloodstream (bacteremia), crosses the blood-brain barrier (BBB), and enters the central nervous system (CNS), where the bacteria contact the meninges. Although GBS is known to invade the BBB, subsequent interaction with astrocytes that physically associate with brain endothelium has not been well studied.

**Methodology/Principal Findings:**

We hypothesize that human astrocytes play a unique role in GBS infection and contribute to the development of meningitis. To address this, we used a well- characterized human fetal astrocyte cell line, SVG-A, and examined GBS infection *in vitro*. We observed that all GBS strains of representative clinically dominant serotypes (Ia, Ib, III, and V) were able to adhere to and invade astrocytes. Cellular invasion was dependent on host actin cytoskeleton rearrangements, and was specific to GBS as *Streptococcus gordonii* failed to enter astrocytes. Analysis of isogenic mutant GBS strains deficient in various cell surface organelles showed that anchored LTA, serine-rich repeat protein (Srr1) and fibronectin binding (SfbA) proteins all contribute to host cell internalization. Wild-type GBS also displayed an ability to persist and survive within an intracellular compartment for at least 12 h following invasion. Moreover, GBS infection resulted in increased astrocyte transcription of interleukin (IL)-1β, IL-6 and VEGF.

**Conclusions/Significance:**

This study has further characterized the interaction of GBS with human astrocytes, and has identified the importance of specific virulence factors in these interactions. Understanding the role of astrocytes during GBS infection will provide important information regarding BBB disruption and the development of neonatal meningitis.

## Introduction

*Streptococcus agalactiae*, also known as Group B *Streptococcus* (GBS), is the leading cause of neonatal meningitis. Meningitis is defined by an inflammation of the protective membranes covering the brain and spinal cord, known collectively as the meninges. Rates of GBS meningitis in neonates have been estimated in the US and in the UK to be 0.65 and 0.72 per 1000 live births respectively [1, 2]. Despite chemotherapeutic intervention, infection can result in high mortality with just under half of surviving infants suffering permanent neurological damage, including cognitive deficits, blindness, hearing loss, seizures and spastic quadriplegia (Cerebral Palsy), [3–6]. Nine different GBS serotypes (Ia, Ib, II-VIII) have been identified and are grouped based on antigenic differences in the structure of the capsule polysaccharide [7]. Three of the nine GBS capsular serotypes (Ia, III and V) have been linked to a majority of neonatal GBS related meningitis [8]. Other GBS virulence factors have been shown to contribute to experimental meningitis including the β-hemolysin/cytolysin (β-h/c) [9], *iagA*, which is required for proper anchoring of lipoteichoic acid (LTA) to the cell wall [10], HvgA [11] and surface proteins that promote interaction with extracellular matrix components such as serine rich repeat protein, Srr1 [12, 13], streptocococcal fibronectin binding protein, SfbA [14], and the pilus tip adhesin, PilA [15].

The development of neonatal GBS disease begins when the bacteria successfully colonize the vaginal epithelium of a pregnant mother. This involved multiple steps before and after birth, which includes bacterial penetration of the placental membranes or inhalation of infected fluids containing GBS. Bacterial meningitis occurs when GBS leaves the bloodstream, breaches the endothelial blood-brain barrier (BBB) and replicates within cerebral spinal fluid (CSF), provoking an overwhelming host inflammatory response [16]. The BBB is primarily composed of a single layer of specialized brain microvascular endothelial cells (BMEC), and together with astrocytes, pericytes, neurons and the extracellular matrix, constitute the neurovascular unit (NVU)[17]. The BBB functions to protect the brain from circulating toxins and microbial infection by maintaining extremely tight intercellular junctions that comprise gap, adherens, and/or desmosomal junctions that link cells together and prohibit pinocytosis [18, 19]. GBS penetration of the BBB involves a complex interplay between the GBS cell surface components and the endothelial cells of the BBB, however, the mechanism(s) by which GBS crosses the BBB and engages the NVU are not well understood. Astrocytes encircle BMEC with their pseudopodia and maintain direct contact with cerebrospinal capillaries. Several studies indicate that astrocytes up-regulate and maintain BBB functions [20–22] and are predicted to have an essential role in protection against invasion by GBS and other microbes [22, 23]. Many studies use human BMEC (hBMEC) as an *in vitro* model to examine how GBS interactions enable endothelial penetration [3, 9, 10, 24]. However, while one study has demonstrated that GBS interacts directly with human fetal astrocytes [25], the nature of these interactions have not been thoroughly investigated. We hypothesize that astrocytes play a unique role in GBS infection and the development of meningitis. In the current study, we examined the capacity of GBS to attach, invade, survive intracellularly, and induce cytokine transcription in a human astrocyte cell line. We further identify GBS surface expressed factors that contribute to these interactions.

## Materials and Methods

### Bacterial strains and growth condition

The following wild-type clinical strains of *Streptococcus agalactiae* GBS were used in this study: COH1 (ATCC BAA 1176), NCTC 10/84 (ATCC 49447), A909 (ATCC 1138), H36B (ATCC BAA 1174), NEM 316 (ATCC 12403), 515 [26]. The following mutant strains of COH1 and NCTC 10/84 were previously generated as described: Δ*cylE* [27], Δ*iag* [10], HY106 [28] [9], Δ*pilA* and Δ*pilB* [18], Δ*srr1* [12], and Δ*sfbA* [14]. The COH1 strain expressing GFP, COH1-GFP, was generated as described [14]. The streptococcal species *Streptococcus gordonii* M99 strain [29] was used as a control. All bacteria were grown in Todd-Hewitt broth (THB) (Hardy Diagnostics, Santa Maria, CA) at 37°C and to early to mid log-phase, equivalent to an optical density of 0.4 measured at 600 nm wavelength which represents ~1 x 10^8^ CFU (Colony Forming Unit)/ml. For antibiotic selection, 2 μg / mL chloramphenicol (Sigma) and 5 μg / mL erythromycin (Sigma) was incorporated in the growth medium when required.

### Cell culture

The human astrocyte cell line, SVG-A was generously provided by Dr. Walter J. Atwood (Brown University, Providence, RI). SVG-A cells were derived from human fetal glial cells transformed with an origin-defective simian virus 40 (SV40) mutant [30]. Cells were grown in DMEM/F12 medium (DMEM/Ham’s F12 50/50) (Mediatech Inc., Manassas, VA.) supplemented with 10% heat-inactivated fetal bovine serum (FBS) and 100X penicillin-streptomycin antibiotic (0.003 units of penicillin and 0.01mg streptomycin/ml) at 37°C and 5% CO_2_. Prior to bacterial infection SVG-A cells were seeded on 24-well plates coated with 1% rat-tail collagen (Fisher Scientific, Pittsburgh, PA) until they reached 90–100% confluency before bacteria were added.

### Bacterial infection assays

GBS adherence, invasion, and intracellular survival assays of SVG-A were conducted as described previously [3]. Briefly, cells were grown to confluency in 24-well tissue culture plates and washed prior to bacterial infection. Bacteria were grown to mid-log phase and added at a multiplicity of infection (MOI) of 0.1, 1 or 10 as indicated for adherence, invasion, and intracellular survival assays. For adherence assays, after 2 hours of incubation, cells were washed 6 times with PBS. Cells were removed from plates by adding 0.025% trypsin-EDTA and then lysed with 0.025% Triton X-100. Lysate was serially diluted and plated on THB agar plates to quantify adherent colony forming units (CFU). Total adherent CFU was calculated as (total CFU recovered/total CFU of original inoculum)×100%. The original inoculum was ~ 1 X 10^5^ CFU. To quantify invading bacteria, cells were incubated with GBS for 2 hours, monolayers washed 3 times with PBS, treated with media containing antibiotics, and incubated for an additional 2 hours for invasion assays, or as given for survival assays. Cells were washed 3 times with PBS, lysed as described above, and viable intracellular GBS determined by serial dilution plating as quantified above.

In indicated experiments the actin polymerization inhibitor cytochalasin D was used to analyze the mechanism of GBS invasion of SVG-A cells. Cytochalasin D (Sigma-Aldrich, St. Louis, MO) was reconstituted at a concentration of 5 mg/ml in DMSO and cells were pretreated with 1, 2, or 5 μg/ml cytochalasin D or DMSO vehicle control for 30 min at 37°C and 5% CO_2_ prior to addition of GBS. Intracellular CFU was recovered and assessed as described above. *Streptococcus gordonii*, a bacterium not associated with CNS disease, was used as a control at an MOI of 1, to assess adherence and invasion compared to clinically relevant GBS isolates.

### Fluorescence Microscopy

To ensure that SVG-A cells represent a homogenous population of astrocytes, cells were stained with APC mouse anti-human CD44, Pgp-1 Ly24 (PharMingen-A Becton Dickinson Co., San Jose, CA) and anti- glial fibrillary acidic protein (GFAP) antibody (Abcam Inc., Cambridge, MA) in 1X PBS at a 1:1000 dilution. DAPI (Vector Laboratories, Burlingame, CA) was used to stain the nucleus. Cells were then fixed with the 4% paraformaldehyde, immunostained and then visualized. To visualize GBS cellular invasion SVG-A cells were grown on collagen-coated glass cover slips to confluence, GBS expressing green fluorescent protein (GFP), COH1-GFP, bacteria were added at an MOI of 10 or 50 and incubated for various times at 37°C. Cell Mask deep red plasma membrane stain (Life Technologies, Grand Island, NY) according to the manufacturers protocol and DAPI nuclear stain were used to visualize intracellular GBS. After processing, coverslips were mounted using Cytoseal-60 (Thermo Scientific, Waltham, MA). Images and Z-stacks were captured using a Zeiss Axio Observer.Z1 inverted microscope using Plan-Apo 63x oil objective equipped with an Axiocam MRm camera device. Axiovision 4.0 software was used to generate images.

### Transmission electron microscopy

SVG-A cells were grown in 24-well plates coated with collagen to confluence, and GBS was subsequently added to monolayers as described above. Following incubation, monolayers were washed with 1 X PBS and collected by centrifugation. Pellets were fixed in 2% gluteraldehyde, 4% formaldehyde in 0.1 M cacodylate buffer at pH 7.4 for 2 h at room temperature. Pellets were then fixed again in 1% osmium tetroxide (OsO_4_) in 0.1M cacodylate buffer for 1 h. After rinsing with water, samples were dehydrated with increasing series of concentrations of ethanol and left on a rotator overnight in a 50% solution of Spurr resin and acetone. The next day samples were placed in 100% Spurr and placed on a rotator for several hours before placing in fresh 100% Spurr again and polymerized in oven for 24h at 60° C. The sample blocks were sectioned at 50 nm on a Leica ultramicrotome, and picked up on formvar-coated copper grids. Grids with sections were stained with uranyl acetate and lead citrate, viewed using a Tecnai-12 (FEI) transmission electron microscope and photographed using a Zeiss 215 side mount digital camera. Images were generated using AMT image capture software. Adobe Photoshop was then used to process individual images and Illustrator was used to compile the images into panels. The TEM images shown represent magnifications of 11,000X (panels a, b, d and e) and insets were magnified at 30,000X (panels c and d), respectively.

### Reverse transcription and real-time quantitative PCR and ELISA

To detect SVG-A cytokine transcripts, cell monolayers were washed twice with PBS following bacterial incubation, and then RPMI 1640 plus 10% FBS was added. SVG-A cells were grown in 24-well plates coated with collagen to confluence, and GBS was subsequently added at MOI 50 bacteria per cell (50:1) to monolayers as described above and incubated at 37°C in 5% CO_2_ for 2,4,6,8, and 12 hours. After infection for the indicated times, cells were rinsed with 1X PBS and total RNA was isolated using the RNeasy miniprep kit (Qiagen Inc., Valencia, California, USA). Total RNA was digested with DNase I to remove any contaminating genomic DNA. Approximately 1 μg of total RNA was utilized for double-stranded cDNA synthesis using the Superscript Choice System (Invitrogen Corp., San Diego, CA). First-strand synthesis and real-time PCR were performed using TaqMan Universal MasterMix SYBR Green (Applied Biosystems, Foster City, CA) and primer sets for IL-1β, IL-6, IL-8, and VEGF genes. The expression level of GAPDH housekeeping gene was used as a control and fold change was reported using the comparative Ct method. Real-time quantitative PCR (RT-qPCR) was performed using the following primer sets (Integrated DNA Technologies, San Jose, CA): IL-6 forward primer 5'-GGA GAC TTG CCT GGT GAA AA-3' and IL-6 reverse primer 5'-CAG GGG TGG TTA TTG CAT CT-3'; IL-1β forward primer 5'-GAC ACA TGG GAT AAC GAG GC-3' and IL-1β reverse primer 5'-ACG CAG GAC AGG TAC AGA TT-3'; IL-8 forward primer 5'- AGC TCT GTG TGA AGG TGC AG -3' and IL-8 reverse primer 5'- AAT TTC TGT GTT GGC GCA GT -3’; VEGF forward primer 5'-ATC TGC ATG GTG ATG TTG GA-3' and VEGF reverse primer 5'-GGG CAG AAT CAT CAG GAA GT-3'; GAPDH forward primer 5'-GAA GGT GAA GGT CGG AGT CAA CG-3' and GAPDH reverse primer 5'-TCC TGG AAG ATG GTG ATG GAA-3’.

For ELISA SVG-A cells were grown in 12-well plates to confluence, and GBS was subsequently added at a MOI 50 bacteria per cell (50:1) to monolayers and incubated at 37°C in 5% CO_2._ Following incubation at indicated time points, cells were washed three times with 1X PBS and collected by addition of 100μL RIPA protein extraction buffer and scratching with a pipette tip for 30 seconds. The concentration of IL-8 was determined in cell lysates using the human CXCL8/IL-8 Quantikine ELISA kit (R&D Systems, Minneapolis, MN) according to manufacturer's instructions.

### Statistical analysis

GraphPad Prism version 6 was used for statistical analysis (GraphPad Software, Inc., La Jolla, CA). Differences between different groups were evaluated using ANOVA with Dunnett’s, Tukey’s or Bonferroni’s multiple comparisons post-test or Unpaired, two tailed t test as indicated. Statistical significance was accepted at *P<*0.05.

## Results and Discussion

### GBS Adheres to and Invades Human Astrocytes

**Fig 1:**
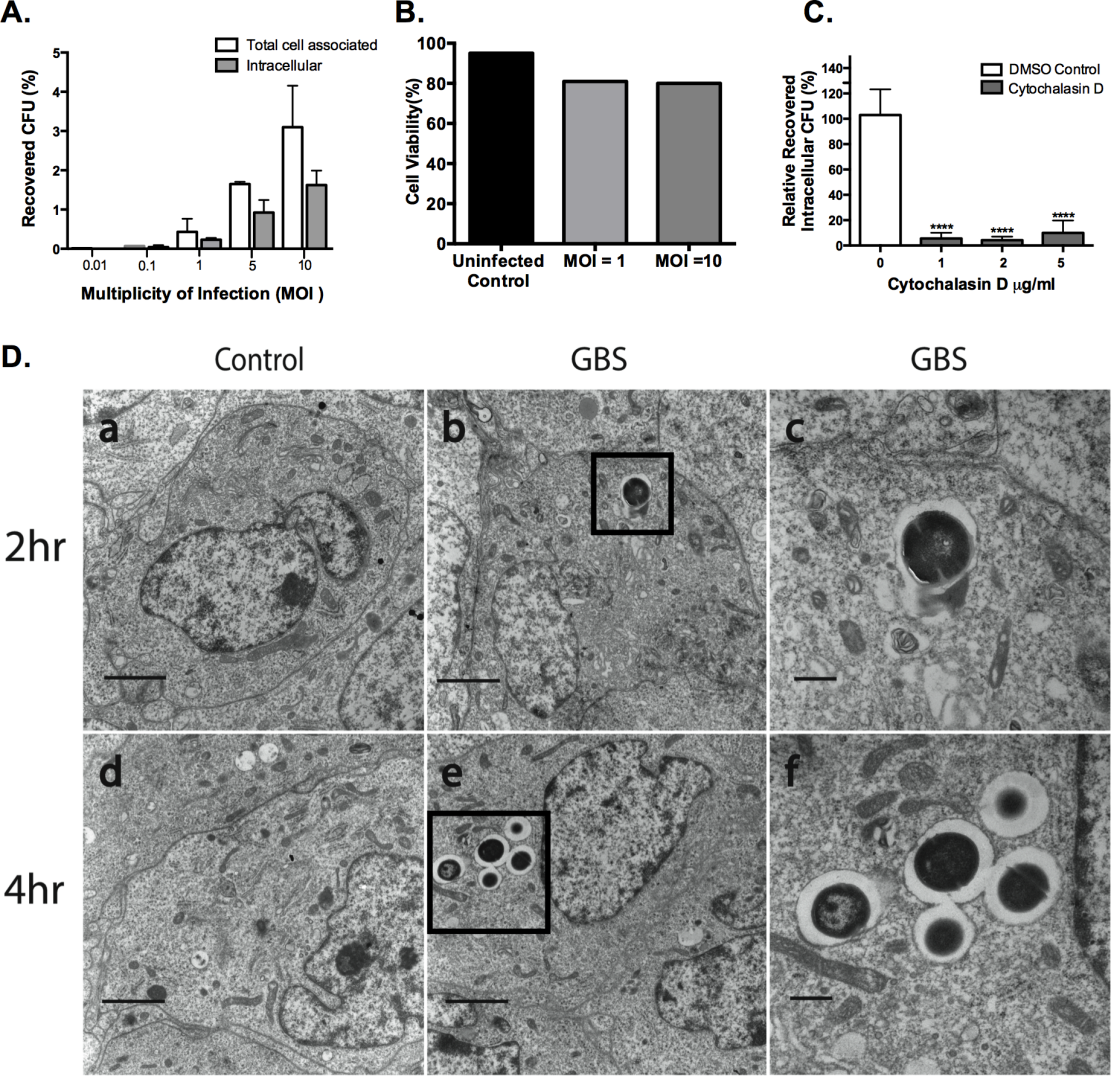
Interaction of GBS with human SVG-A astrocytes. A, confluent monolayers of SVG-A cells were infected with GBS strain COH1 at the indicated MOI for 30 minutes for adhesion and 2 h for invasion assays and the extent of bacterial adherence and invasion were quantified as described in Materials and Methods. The data are expressed as the percentage of recovered CFU/initial inoculum. All experiments were performed in triplicate and repeated in at least three independent experiments. B, SVG-A cells were infected with COH1 at an MOI of 1 and 10 for 24 hours and percent cell viability determined by trypan blue dye exclusion and compared to an uninfected control. C, SVG-A cells were pretreated in media alone, DMSO vehicle control or cytochalasin D (Cyt D) at 1 to 5 μg/ml for 30 min at 37°C and 5% CO_2_. Cells were then incubated with COH1 at an MOI of 1 for 2 h and intracellular GBS was quantified. The data shown represents the percentage of bacterial uptake after cytochalasin D treatment relative to untreated control. Significance was determined by one-way ANOVA using tukeys multiple comparison test, P < 0.0001 (****). D, Transmission electron microscopic (TEM) images of SVG-A cells that were either uninfected (control) or infected with GBS COH1 at an MOI of 10. For TEM scale bars for panels A, B, D, and E are 2um and for C and F are 500nm.

Given that adhesion of GBS to host cells is required for invasion and overt infection, we examined the specific conditions of GBS interactions with human astrocytes utilizing the SVG-A cell line. We observed that the SVG-A cells line, which is of human astroglial cell origin [30], exhibited homogeneous morphology and robust expression of GFAP and CD44 as expected (data not shown). To determine the extent of GBS surface-adherence and invasion, confluent SVG-A cells were infected with increasing MOIs of WT GBS COH1, the hypervirulent serotype III and sequence type (ST)-17 strain that is strongly associated with neonatal meningitis [31]. The amount of surface adherent and invasive GBS increased with increasing MOI (Fig 1A). The percentage of adherent and intracellular bacteria ranged from 0.01%–4.2% for total cell associated GBS and from 0%–2.1% for invasion compared with the original inoculum (Fig 1A) At the standard inoculum used (MOI 1–10), approximately 50% of total SVG-A-associated GBS had invaded the intracellular compartment within the 2 h incubation period, suggesting efficient uptake of surface-adherent organisms. The weakly hemolytic COH1 strain did not appear to be cytotoxic even at MOI of 10, as SVG-A monolayers remained intact as assessed by trypan blue exclusion staining (Fig 1B). A key event leading to bacterial internalization of host cells involves actin polymerization. To examine if this host cellular process is important for GBS invasion of human astrocytes, SVG-A cells were treated with cytochalasin D to inhibit actin polymerization. Confluent monolayers of SVG-A cells were treated with varying concentrations of cytochalasin D or DMSO vehicle control for 30 mins at 37°C with 5% CO_2_. After cytochalasin D pretreatment, SVG–A cells were then infected with GBS COH1 strain at an MOI of 1 for 2 h. Monolayers were then washed and processed to determine the amount of internalized bacteria as described in Materials and Methods. GBS invasion was similar in non-treated or DMSO-treated controls (data not shown). We observed a decrease in GBS invasion in astrocytic cells in the presence of cytochalasin D compared to the DMSO treated control (Fig 1C). These results suggest that GBS requires actin polymerization and cytoskeletal rearrangement of the host cell for uptake into astrocytes. This finding is consistent with previous reports demonstrating the requirement of actin polymerization and cytoskeletal rearrangement for the uptake of bacteria into host cells [32]-[33],[3].

GBS invasion of human astrocytes was confirmed and visualized using transmission electron microscopy (TEM). SVG-A cells were either mock infected or infected with WT GBS COH1 at an MOI of 10 for 2 h at 37°C and 5% CO_2_. Cells were then treated with antibiotics for 2 or 4 h to remove extracellular bacteria and then processed for TEM. GBS was observed within membrane bound intracellular vesicles within astrocytes, and under these conditions the SVG-A morphology was not affected by GBS infection (Fig 1D). Together these data confirm previous reports and demonstrate that GBS has the capacity to adhere to and invade human astrocytes. Interestingly, after 4 h of antibiotic treatment we observed an increase in the number of vesicles containing intracellular GBS present in SGV-A compared to the 2 h time point (Fig 1D, panels b, c, e and f). In addition, several bacteria appear to have septa that are indicative of bacterial division and vesicular-like structures appear to be fusing (Fig 1D, panels c and f). Autophagosomes are frequently observed fusing with endosomal or lysosomal vesicles and are double membrane structures [34]. Reports show that in TEM plastic sections, the double membranes can be so close to each other that it is not possible to see them as separate membranes and sometimes the autophagosome limiting membrane does not have any contrast in thin sections [35]. Although, the internal vesicles containing GBS may be reminiscent of autophagosomes [36–38], we have not yet defined the nature of these compartments.

### Analysis of GBS—Astrocyte Interaction

**Fig 2:**
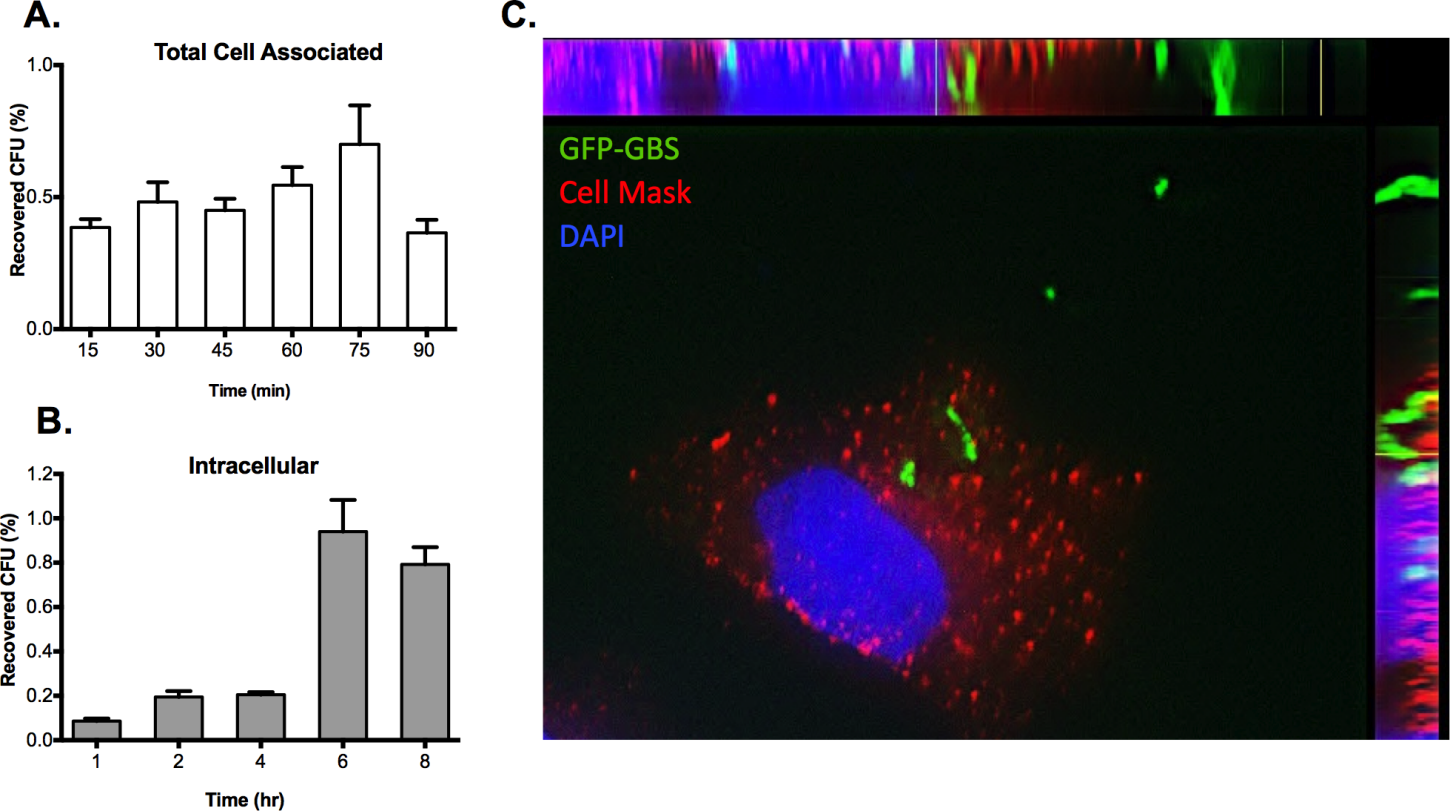
GBS adhesion, invasion and intracellular survival of SVG-A cells. A and B, Confluent monolayers of SVG-A cells were incubated with GBS, COH1 wild-type strain, at an MOI of 1 for various times at 37°C, 5% CO_2_ and adhesion and invasion was examined as described in Materials and Methods. The data shown are a representative of 3 independent experiments performed in triplicate. Incubation at shorter and longer times resulted in a similar level of adhesion and invasion of COH1 to SVG-A cells and was not statistically significant as determined by one-way ANOVA and Tukey's multiple comparison tests. C, SVG-A cells were infected with COH1 expressing GFP at an MOI of 10 and incubated for 8 h at 37°C. Cells were fixed and processed for fluorescence microscopy and stained with Cell Mask (red) to visualize the plasma membrane or with DAPI (blue) to visualize the nucleus. The image is representative of many images from 2 independent experiments.

Next, we evaluated the extent of GBS wild-type COH1 adherence to and invasion of SVG-A cells over time. SVG-A cells grown to confluence were incubated with COH1 at an MOI of 1 for 0 to 90 min at 37°C and 5% CO_2_ and total cell associated GBS was examined. Interestingly, the extent of GBS adherence to SVG-A cells did not significantly change over the 90 min time-course, suggesting that the levels of adherent bacteria remains quantitatively consistent over this time course (Fig 2A). In contrast, invasion of SVG-A cells by COH1 at an MOI of 1 markedly increased from 4 to 6 h post infection and appears to reach steady-state following 8 h post infection (Fig 2B). These results suggest that SVG-A cells are susceptible to invasion for many hours and this results in a substantial amount of GBS accumulation that appears to be maximal at 6 and 8 h post infection (Fig 2B). We further used fluorescence microscopy to visualize a COH1-GFP persisting inside SVG-A cells at 8 h as described in Materials and Methods (Fig 2C).

**Fig 3:**
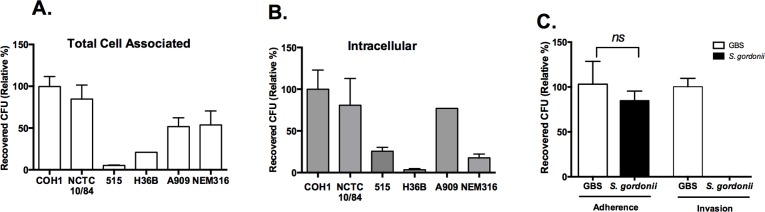
Clinically relevant isolates of GBS adhere and invade, but non-GBS, bacteria fail to invade human astrocytes. A and B, Wild-type GBS serotypes (Ia, Ib, III, and V), were incubated with confluent SVG-A cells at an MOI of 1 for 30 minutes for adhesion and 2 h for invasion assays and the levels of both surface-adherence and invasion were quantified. These data are expressed as the relative percentage of clinical isolates compared to COH1, a representative experiment of at least 3 independent experiments performed in triplicate is shown. C, SVG-A cells grown to confluence were infected with GBS COH1 at an MOI of 1 or the non-GBS strain, *S*. *gordonii* for 30 min for adhesion or 2 h for invasion assays. An unpaired, two tailed t-test with Welch’s correction was used to compare S. *gordonii* to COH1. Data are expressed as the relative percent adherence or invasion and are representative of 3 independent experiments performed in triplicate or quadruplicate.

Next, we examined whether human astrocytes were susceptible to invasion by other clinically relevant GBS isolates representing diverse serotypes as described in Table 1. The GBS capsular serotypes commonly associated with bloodstream infections in newborns are Ia, Ib, II, III, and V, with type III accounting for a disproportionate amount of meningeal infection [3, 39]. We compared invasion of SVG-A cells by two clinically dominant GBS clinical isolates, COH1 and NCTC 10/84 (serotype III and V respectively) to the clinical isolates belonging to other capsular serotypes which include strains 515 (Ia), H36B (Ib), A909 (Ia) and NEM316 (III). Our data indicate that all GBS strains exhibited varying levels of adherence and invasion compared to COH1 (Fig 3A and 3B). Interestingly the hyper-hemolytic strain NCTC 10/84 was equally adherent and invasive as COH1, suggesting that toxin production may not impact these interactions. In contrast, GBS strains 515, H36B, and NEM316 exhibited minimal adherence and/or invasion of SVG-A cells compared to COH1 (Fig 3A and 3B). These findings demonstrate that GBS isolates are able to adhere and invade astrocytes to varying degrees, which may be due to differences in cell surface expressed determinants resulting in divergent affinities or capacities for binding to human astrocytes. The variable invasiveness exhibited by the different GBS strains has been previously observed for other cell types such as human vaginal epithelial cells [40], human pulmonary alveolar epithelial cells (A549)[41], human umbilical vein endothelial cells (HUVEC)[42], and human brain endothelial cells (hBMEC) [3, 43]. Interestingly, there was no correlation between the ability of GBS to adhere versus invade astrocytic cells (Fig 3A and 3B).

**Table 1:** Bacterial strains used in the study.

**Organism**	**ATCC Reference No.**	**Serotype**	**Comments**	**Reference**
*S*. *agalactiae*				
COH1	BAA 1176	III	WT, highly encapsulated	[77]
HY106	----	III	COH1, capsule mutant	[9, 28]
COH1∆*cylE*	----	III	ß-h/c deficient	[27, 41]
COH1∆*iagA*		III	Lack LTA anchor	[10, 78]
COH1∆*sfbA*		III	SfbA deficient	[14]
NCTC 10/84	ATCC: 49447; 1169-NT1	V	WT, hyper hemolytic	[79]
NCTC 10/84 ∆*cylE*	----	V	ß-h/c deficient	[27]
NCTC 10/84 ∆*pilA*	----	V	Lacks pilus tip adhesin	[18]
NCTC 10/84 ∆*pilB*	----	V	Lacks pilus backbone	[18, 78]
NCTC 10/84 ∆*srr-1*		V	Lacks Serine Rich Repeat Protein	[12]
A909	BAA: 1138	Ia	WT	[80]
515	----	Ia	WT	[81],[82]
H36B	BAA: 1174	Ib	WT	[81]
NEM 316	ATCC: 12403	III	WT	[82]
*S*. *gordonii M99*	----	Type 37 capsule polysaccharide	WT	[83, 84]

WT, wild-type

We next assessed whether astrocyte adherence and invasion was specific by examining another streptococcal species, *Streptococcus gordonii*, a Gram-positive oral pathogen. Our results demonstrated that *S*. *gordonii*, exhibited similar levels of attachment to SVG-A cells compared to COH1, but was not able to invade these cells (Fig 3C). Together these data suggest that there may be unique factors expressed by GBS that facilitate adherence and invasion that are essential to trigger bacterial entry into astrocytes.

### Contribution of GBS virulence factors to astrocyte interactions

**Fig 4:**
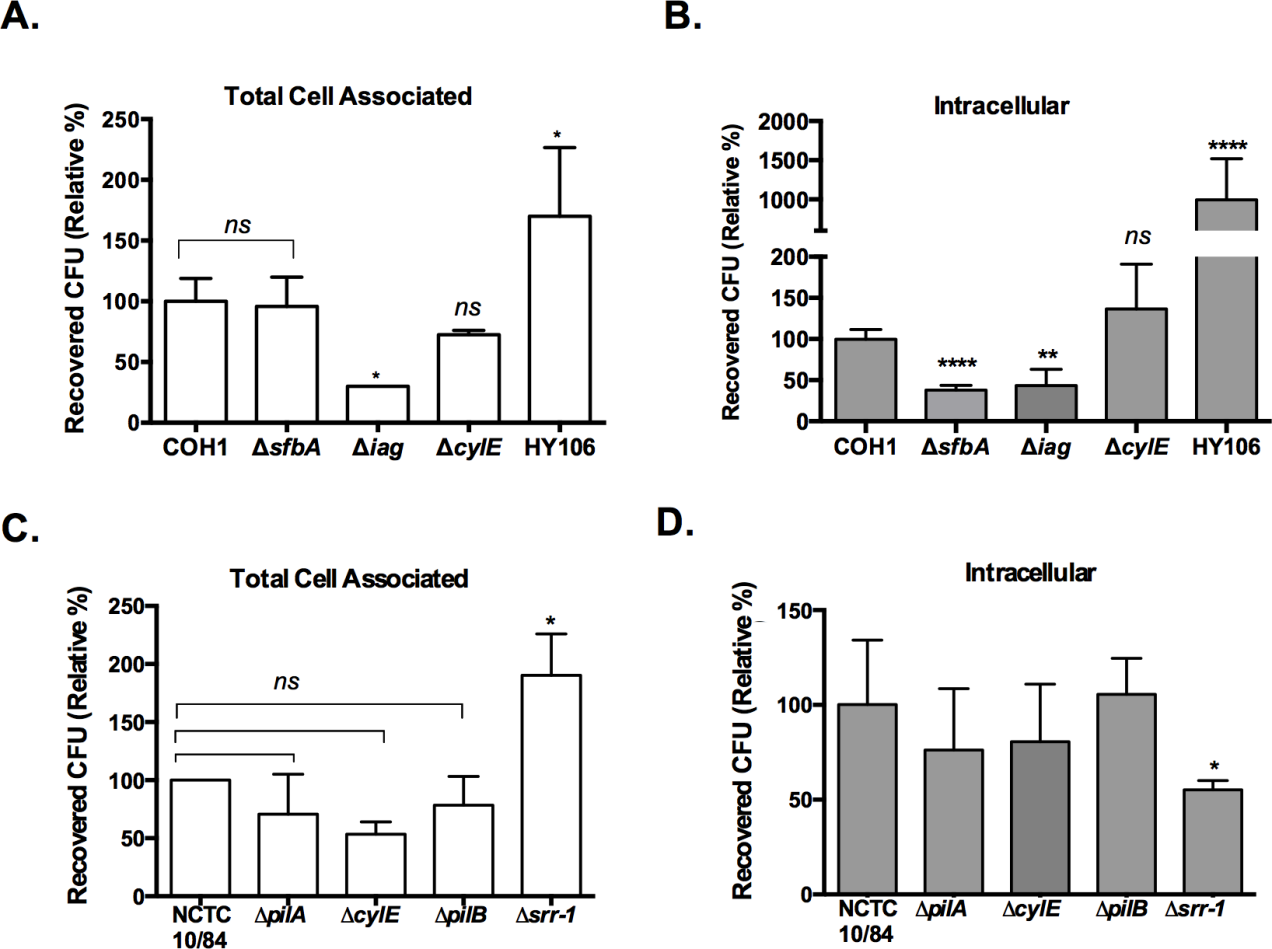
Role of GBS surface determinants in SVG-A interaction. A and B, SVG-A cells grown to confluence were infected at an MOI of 1 with wild type strains COH1 or NCTC10/84 and respective isogenic mutants. SVG-A cells were infected for 30 min for adhesion or 2 h for invasion assays, bacteria were recovered and quantified as described in Materials and Methods. A representative experiment of at least 3 independent experiments performed in triplicate is shown. Data are expressed as the percentage of recovered CFU for GBS mutants relative to that of the wild type parental strain. Data were analyzed by unpaired, two-tailed t-test. (*) = P< 0.05, (**), P< 0.01 and (****), P< 0.0001.

The interaction of bacterial surface components with host cell receptors to initiate cell and tissue penetration is a prerequisite for development of severe invasive disease. GBS surface expressed factors shown to promote GBS attachment and/or invasion of different host cell types include the invasion associated gene, *iagA* [10], capsule [9, 28], β-hemolysin/cytolysin (β-h/c) [9], pili [18], serine-rich repeat glycoproteins, including Srr-1 [12], fibrinogen-binding proteins [44, 45], and a recently identified fibronectin binding protein, SfbA [14]. To investigate the importance of certain bacterial components in the adherence and invasion of human astrocytes, wild-type GBS and isogenic mutants were used (see Table 1). Confluent monolayers of SVG-A cells were infected with an MOI of 1 of wild type COH1 and NCTC10/84 strains and indicated isogenic mutants. The Δ*iagA* mutant exhibited reduced levels of adherence and invasion compared to the parental COH1 strain (Fig 4A and 4B). The *iagA* gene encodes a glycosyltransferase that is required for the synthesis of glycolipid diglucosyldiacylglycerol, a cell membrane anchor for lipoteichoic acid (LTA) [10]. Previous studies have shown that *iagA* function is important to properly anchor LTA in the GBS cell wall, and that LTA expression on the GBS surface plays a role in bacterial interaction with BBB endothelium and the pathogenesis of neonatal meningitis [10]. Thus our data suggest that similar interactions are important for GBS attachment and entry into astrocytic cells. Our results also demonstrate that fibronectin binding protein, SfbA, is important for invasion into astrocytes, although it did not contribute to GBS attachment (Fig 4A and 4B). These results are similar to that observed for the role of SfbA in the interaction of GBS with host endothelial and epithelial cells [14]. In contrast, the unencapsulated HY106 strain displayed an increase in both adherence and invasion (Fig 4A and 4B). This is not completely unexpected as the polysaccharide capsule likely masks bacterial surface determinants that may promote interaction with the host cell.

Interestingly, isogenic mutants in both strain backgrounds that were deficient in β-h/c (Δ*cylE*) exhibited similar levels of adherence and invasion to parental strains (Fig 4). This suggests that toxin production does not contribute to cellular interactions and is consistent with our results above comparing the interactions of COH1 (weakly hemolytic) to NCTC 10/84 (hyper-hemolytic). Additionally, previous studies also demonstrated that toxin production did not impact GBS adherence to astrocytic cells [25].

Proteins targeted for cell surface expression in GBS are predicted to share a C-terminal sequence (L/IPXTG) for sortase recognition and anchoring to the Gram-positive cell wall. Several cell wall anchored proteins promoting GBS interaction with brain microvascular endothelial cells have been identified and characterized [23]. An important determinant recently implicated in fibrinogen binding and BBB interaction are the GBS serine rich repeat (Srr) glycoproteins [12] Srr proteins have a highly conserved domain organization, including a long and specialized signal sequence, two extensive serine-rich repeat regions that undergo glycosylation, and a typical LP(X)TG cell wall anchoring motif. We observed that Srr-1 contributed significantly to astrocyte entry, even though the Srr-1 deficient mutant appeared to be more adherent (Fig 4C and 4D). These results are similar to what has been reported previously in BBB endothelium [12], demonstrating that GBS Srr-1 contributes to penetration of different host cell barriers.

Additional cell wall anchored proteins in GBS make up pilus structures. Pili are flexible appendages on the bacterial surface that mediate infection, including adhesion to host cells, DNA transfer and biofilm formation [46–48]. Cell surface pili have been described in GBS and other streptococcal pathogens [49–51]. The genes encoding pili in GBS are located within two distinct loci, and generally consist of three genes that encode LPXTG motif-carrying proteins corresponding to the major pilus subunit (PilB), two ancillary proteins (PilA and PilC; [52]). We examined the ability of isogenic mutants lacking either PilA or PilB to interact with the SVG-A cell line. Surprisingly we did not observe a difference in the ability of PilA or PilB deficient mutants to interact with astrocytes compared to the WT parental strain (Fig 4C and 4D). Thus, SVG-A cells may also recognize GBS independent of typical bacterial attachment mechanisms. One of the functional roles of astrocytes is to clean up cell debris in order to limit tissue damage following injury or infection of the brain [53, 54]. Astrocytes possess MEGF10 (multiple EGF-like-domains 10) and P2X7 receptors, which are known to be phagocytic receptors [54, 55]. Alternatively, astrocytes have been reported to become activated and proliferate when exposed to bacterial endotoxins and lipopolysaccharides (LPS)[56–58]. It is possible that GBS is recognized and enters astrocytic cells by phagocytosis, but future studies are needed to examine this possibility.

### GBS Intracellular Survival in Human Astrocytes

**Fig 5:**
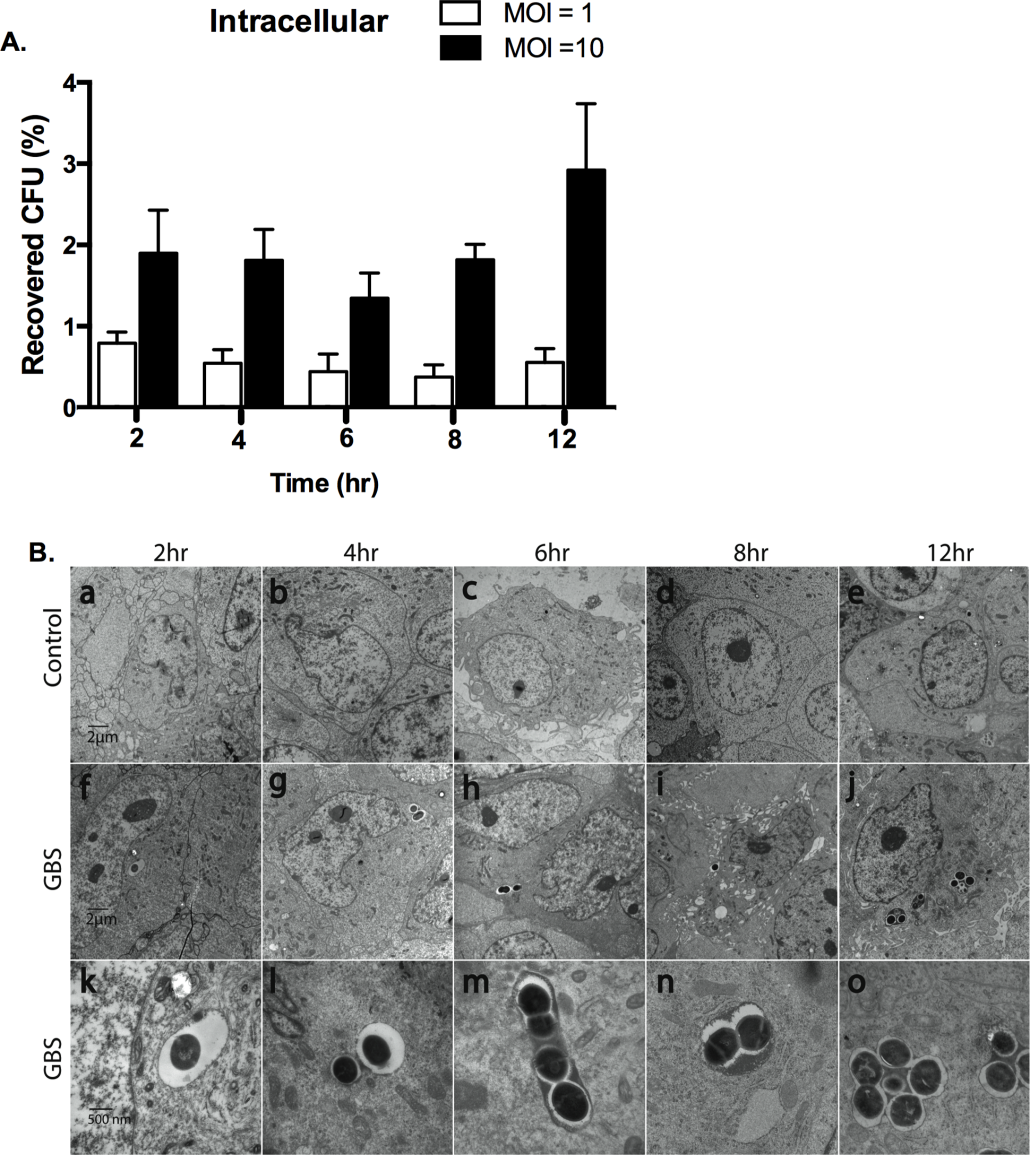
Intracellular survival of wild-type GBS in human astrocytes. A, Wild-type GBS, COH1, intracellular survival assays in SVG-A cells were carried out for up to 12 hr at MOI of 1 and 10. To assess intracellular survival in SVG-A cells over time, intracellular bacteria were enumerated after the addition of antibiotics (gentamicin (50 μg/ml) and penicillin (25 μg/ml) from 2 to 12 h time points. B, Transmission electron micrographs demonstrating GBS (COH1) intracellular survival over time in SVG-A cells at magnifications (6,000X for A-J, and 30,000X for K-O). All assays were performed in triplicate or quadruplet and repeated at least three times.

We sought to determine if GBS may survive intracellularly and replicate within astrocytes for longer periods of time following intracellular invasion. To test this, we recovered intracellular GBS following antibiotic treatment to kill extracellular bacteria over time as described in Materials and Methods. We observed that GBS was able to survive for up to 12 h post infection regardless of the initial MOI used (Fig 5A). GBS infected cells were also examined by TEM over a 2 to 12 h time-course (5B). This was done to assess cell morphology after infection over time and to confirm our findings. Intriguingly, dividing forms of GBS COH1 were observed inside SVG-A cells within large endocytic vacuoles after 2 h post infection (Fig 5B, panels f-o). In addition, the number of intracellular GBS COH1 detected increased considerably from 6 h to 12 h (Fig 5B, panels f-o). We were also able to capture images of bacteria with septa, which are indicative of dividing bacteria [59, 60], along with electron dense material around or between these bacteria (Fig 5B, panels h-o). This electron dense material was only observed at the later times and may be evidence of a bacterial protein or biofilm being secreted. Some of internal compartments also contained an increase of number of bacteria or bacterial aggregates (Fig 5B, panels g-o), whereas other internal compartments containing bacteria appear to be fused together (Fig 5B, panels h-j and m-o). These data indicated that GBS persists within SVG-A cells for an extended period of time following invasion and possibly undergo replication and fusion within intracellular compartments. However our recovery and quantification of viable cfu (Fig 5A) suggests that there is no net replication over time.

### Astrocyte activation during GBS infection

Proinflammatory cytokines IL-1β and IL-6 are major early response mediators that often trigger other cytokines and chemokines, arachidonic acid metabolites, and reactive nitrogen and oxygen intermediates in response to tissue injure or infection [61–63]. Multiple cell types within the CNS including microvascular endothelial cells, astrocytes, and microglia secrete IL-1β, and IL-6. Increased concentrations of these cytokines have been detected in cerebral spinal fluids taken from patients with acute bacterial meningitis and concentrations of IL-1β are associated with significantly an increase in disease severity [64].

**Fig 6:**
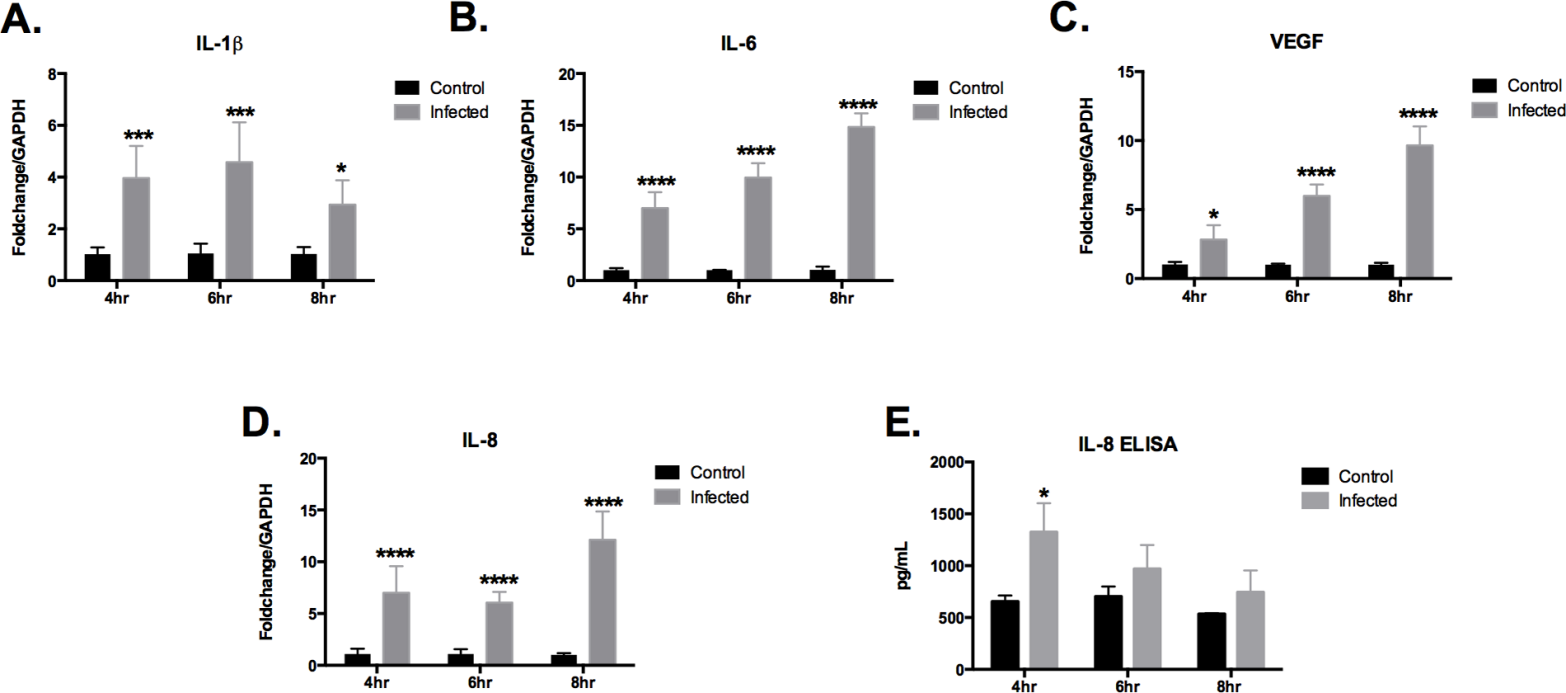
Activation of human astrocytes following GBS infection. A-D, Transcript abundance of IL-1β, IL-6, VEGF, and IL-8 in SVG-A cells was determined using quantitative RT-PCR following infection with GBS COH1 (MOI = 50). Fold change was calculated using GAPDH and then normalized to media controls as described in Materials and Methods. E, Protein production of IL-8 was determined by ELISA as described in Materials and Methods. Data is one representative experiment of at least 2 independent experiments performed in a minimum of 4 replicates. Data was analyzed by two-way ANOVA with Bonferroni’s multiple comparisons post-test. (*) = P< 0.05, (***) = P< 0.001, (****) = P< 0.0001.

Alterations in synthesis and release of cytokines as a host response to GBS infections have previously been shown to weaken barrier integrity (mucosal, epithelial, endothelial) by the upregulation of interleukins 1, 6, 8 (IL-1β, IL-6, IL-8)—[9, 15, 65–69]. We first examined the cytokine production of GBS infected astrocytes. The measurement of pro-inflammatory cytokines IL-1β, IL-6, chemokine IL-8, and vascular endothelial growth factor (VEGF) signaling protein was carried out by quantifying mRNA expression levels as described previously [15]. We investigated these factors due to their role in the host response during GBS infection of brain endothelium and other cell types [9, 66, 67, 70–72]. SVG-A cells were infected with GBS over time as described in Materials and Methods and transcript abundance of IL-1β, IL-6, IL-8 and VEGF were measured and normalized to the expression level of GAPDH housekeeping gene and compared to uninfected controls. All cytokines were significantly induced during GBS infection at all time points examined (Fig 6A–6D). An ELISA was performed to assay IL-8 protein levels, which demonstrated a significant increase in IL-8 protein at 4 hours post GBS infection (Fig 6E). These results are the first to demonstrate that GBS infection induces astrocyte cytokine production, which may contribute to the increase in meningeal inflammation. We hypothesize that once the GBS penetrates brain endothelial cells and gains access to astrocytes, GBS further induces an astrocytic inflammatory response, which could impact endothelial barrier integrity. IL-8 is a potent neutrophil chemokine that is involved in neutrophil activation and recruitment [73] neutrophil migration has been shown to promote BBB permeability during experimental GBS meningitis [15]. Secretion of cytokines and chemokines by astrocytes may allow GBS to penetrate further through the BBB, thus, contributing to the progression of meningitis. Alternatively, this immune response may represent an effective second line of host defense or neuroprotection [74, 75], preventing further penetration of GBS into the CNS. However, subsequent increases in proinflammatory pathways produced after GBS infection, could intensify the permeability or leakiness of the BBB resulting in further exacerbation or the development of meningitis. Interestingly VEGF, which increases brain endothelial permeability when given intravenously, is neuroprotective and reduces BBB leakage after ischemia when given intraventricularly [76]. Further investigation is required to determine the exact role of astrocyte derived VEGF and other proinflammatory cytokines on BBB permeability during GBS meningitis.

In summary we have demonstrated that GBS actively invades and persists in human astrocytes and that various bacterial surface factors promote these interactions. Further astrocytes respond to GBS infection with a robust proinflammatory response, which may impact disease progression. Understanding the role of astrocytes and the NVU during bacterial infection will provide important information regarding BBB disruption and the development of neonatal meningitis.

## References

[1] Heath PT, Balfour G, Weisner AM, Efstratiou A, Lamagni TL, Tighe H, et al Group B streptococcal disease in UK and Irish infants younger than 90 days. The Lancet. 2004;363(9405):292–4. 10.1016/s0140-6736(03)15389-5 14751704

[2] Thigpen MC. Bacterial Meningitis in the United States, 1998–2007. The New England Journal of Medicine. 2011;364(21):2016–25. 10.1056/NEJMoa1005384 21612470

[3] Nizet V, Kim KS, Stins M, Jonas M, Chi EY, Nguyen D, et al Invasion of brain microvascular endothelial cells by group B streptococci. Infection and Immunity. 1997;65(12):5074–81. 9393798 10.1128/iai.65.12.5074-5081.1997 PMC175731

[4] Chin K, Fitzhhardinge P. Sequelae of early-onset group B hemolytic streptococcal neonatal meningitis. J Pediatr. 1985;106(5):819–22. 3889251 10.1016/s0022-3476(85)80365-6

[5] Edwards MS RM, Haffar AA, Murphy MA, Desmond MM, Baker CJ. Long-term sequelae of group B streptococcal meningitis in infants. J Pediatr 1985;106(5):717–22. 3889248 10.1016/s0022-3476(85)80342-5

[6] Libster R, Edwards KM, Levent F, Edwards MS, Rench MA, Castagnini LA, et al Long-term outcomes of group B streptococcal meningitis. Pediatrics. 2012;130(1):e8–15. Epub 2012/06/13. 10.1542/peds.2011-3453 . 22689869

[7] Cieslewicz MJ, Chaffin D, Glusman G, Kasper D, Madan A, Rodrigues S, et al Structural and genetic diversity of group B streptococcus capsular polysaccharides. Infect Immun. 2005;73(5):3096–103. Epub 2005/04/23. 10.1128/IAI.73.5.3096-3103.2005 15845517 PMC1087335

[8] Ferrieri ASP. Neonatal & maternal group B streptococcal infections: A comprehensive review. Indian J Med Res 2004;120:141–50. 15489551

[9] Doran KS, Liu GY, Nizet V. Group B streptococcal β-hemolysin/cytolysin activates neutrophil signaling pathways in brain endothelium and contributes to development of meningitis. Journal of Clinical Investigation. 2003;112(5):736–44. 10.1172/jci200317335 12952922 PMC182187

[10] Doran KS, Engelson EJ, Khosravi A, Maisey HC, Fedtke I, Equils O, et al Blood-brain barrier invasion by group B Streptococcus depends upon proper cell-surface anchoring of lipoteichoic acid. J Clin Invest. 2005;115(9):2499–507. Epub 2005/09/03. 10.1172/JCI23829 16138192 PMC1193870

[11] Tazi A, Disson O, Bellais S, Bouaboud A, Dmytruk N, Dramsi S, et al The surface protein HvgA mediates group B streptococcus hypervirulence and meningeal tropism in neonates. J Exp Med. 2010;207(11):2313–22. Epub 2010/10/20. 10.1084/jem.20092594 20956545 PMC2964583

[12] van Sorge NM, Quach D, Gurney MA, Sullam PM, Nizet V, Doran KS. The group B streptococcal serine-rich repeat 1 glycoprotein mediates penetration of the blood-brain barrier. J Infect Dis. 2009;199(10):1479–87. Epub 2009/04/28. 10.1086/598217 19392623 PMC2674616

[13] Seo HS, Mu R, Kim B, Doran K, Sullam P. Binding of Glycoprotein Srr1 of Streptococcus agalactiae to Fibrinogen Promotes Attachment to Brain Endothelium and the Development of Meningitis. Plos Pathogens. 2012;8(10):1–12. 10.1371/journal.ppat.1002947 PMC3464228 23055927

[14] Mu R, Kim BJ, Paco C, Del Rosario Y, Courtney HS, Doran KS. Identification of a group B streptococcal fibronectin binding protein, SfbA, that contributes to invasion of brain endothelium and development of meningitis. Infect Immun. 2014;82(6):2276–86. Epub 2014/03/20. 10.1128/IAI.01559-13 24643538 PMC4019170

[15] Banerjee A, Kim BJ, Carmona EM, Cutting AS, Gurney MA, Carlos C, et al Bacterial Pili exploit integrin machinery to promote immune activation and efficient blood-brain barrier penetration. Nat Commun. 2011;2:462 Epub 2011/09/08. 10.1038/ncomms1474 21897373 PMC3195231

[16] Kim Y. Brain injury in experimental neonatal meningitis due to group B streptococci. J Neuropathol Exp Neurol 1995;54(4):531–9. 7602326 10.1097/00005072-199507000-00007

[17] Hawkins BT, Davis TP. The blood-brain barrier/neurovascular unit in health and disease. Pharmacol Rev. 2005;57(2):173–85. Epub 2005/05/26. 10.1124/pr.57.2.4 . 15914466

[18] Maisey HC, Hensler M, Nizet V, Doran KS. Group B streptococcal pilus proteins contribute to adherence to and invasion of brain microvascular endothelial cells. J Bacteriol. 2007;189(4):1464–7. Epub 2006/10/17. 10.1128/JB.01153-06 17041051 PMC1797338

[19] Kabanov AV, Gendelman HE. Nanomedicine in the diagnosis and therapy of neurodegenerative disorders. Prog Polym Sci. 2007;32(8–9):1054–82. Epub 2007/01/01. 10.1016/j.progpolymsci.2007.05.014 20234846 PMC2838200

[20] Abbott NJ, Ronnback L, Hansson E. Astrocyte-endothelial interactions at the blood-brain barrier. Nat Rev Neurosci. 2006;7(1):41–53. Epub 2005/12/24. 10.1038/nrn1824 . 16371949

[21] Haj-Yasein NN, Vindedal GF, Eilert-Olsen M, Gundersen GA, Skare O, Laake P, et al Glial-conditional deletion of aquaporin-4 (Aqp4) reduces blood-brain water uptake and confers barrier function on perivascular astrocyte endfeet. Proc Natl Acad Sci U S A. 2011;108(43):17815–20. Epub 2011/10/13. 10.1073/pnas.1110655108 21990350 PMC3203818

[22] Xie L, Poteet EC, Li W, Scott AE, Liu R, Wen Y, et al Modulation of polymorphonuclear neutrophil functions by astrocytes. J Neuroinflammation. 2010;7:53 Epub 2010/09/11. 10.1186/1742-2094-7-53 20828397 PMC2942816

[23] van Sorge NM, Doran KS. Defense at the border: the blood-brain barrier versus bacterial foreigners. Future Microbiol. 2012;7(3):383–94. Epub 2012/03/08. 10.2217/fmb.12.1 22393891 PMC3589978

[24] Shin S, Paul-Satyaseela M, Lee JS, Romer LH, Kim KS. Focal adhesion kinase is involved in type III group B streptococcal invasion of human brain microvascular endothelial cells. Microb Pathog. 2006;41(4–5):168–73. Epub 2006/09/05. 10.1016/j.micpath.2006.07.003 . 16949788

[25] Alkuwaity K, Taylor A, Heckels JE, Doran KS, Christodoulides M. Group B Streptococcus Interactions with Human Meningeal Cells and Astrocytes In Vitro. PLoS ONE. 2012;7(8):e42660 10.1371/journal.pone.0042660 22900037 PMC3416839

[26] Wessels MR, Paoletti LC, Rodewald AK, Michon F, DiFabio J, Jennings HJ, et al Stimulation of protective antibodies against type Ia and Ib group B streptococci by a type Ia polysaccharide-tetanus toxoid conjugate vaccine. Infect Immun. 1993;61(11):4760–6. Epub 1993/11/01. 8406875 10.1128/iai.61.11.4760-4766.1993 PMC281231

[27] Pritzlaff. Genetic basis for the beta-haemolytic/cytolytic activity of group B Streptococcus. Molec Microbiol. 2001;39(2):236–47. 11136446 10.1046/j.1365-2958.2001.02211.x

[28] Yim H. Analysis of the Capsule Synthesis Locus, A Virulence Factor in Group B Streptococci. 1997. 10.1007/978-1-4899-1825-3_234 9331818

[29] Bensing BA, Lopez JA, Sullam PM. The Streptococcus gordonii surface proteins GspB and Hsa mediate binding to sialylated carbohydrate epitopes on the platelet membrane glycoprotein Ibalpha. Infect Immun. 2004;72(11):6528–37. Epub 2004/10/27. 10.1128/IAI.72.11.6528-6537.2004 15501784 PMC523053

[30] Major EO, Miller AE, Mourrain P, Traub RG, de Widt E, Sever J. Establishment of a line of human fetal glial cells that supports JC virus multiplication. Proc Natl Acad Sci U S A. 1985;82(4):1257–61. Epub 1985/02/01. 2983332 10.1073/pnas.82.4.1257 PMC397234

[31] Lamy MC, Dramsi S, Billoet A, Reglier-Poupet H, Tazi A, Raymond J, et al Rapid detection of the "highly virulent" group B Streptococcus ST-17 clone. Microbes Infect. 2006;8(7):1714–22. Epub 2006/07/11. 10.1016/j.micinf.2006.02.008 . 16822689

[32] Clerc PL, Ryter A, Mounier J, Sansonetti PJ. Plasmid-mediated early killing of eucaryotic cells by Shigella flexneri as studied by infection of J774 macrophages. Infect Immun. 1987;55(3):521–7. Epub 1987/03/01. 3546130 10.1128/iai.55.3.521-527.1987 PMC260367

[33] Finlay BB, Rosenshine I, Donnenberg MS, Kaper JB. Cytoskeletal composition of attaching and effacing lesions associated with enteropathogenic Escherichia coli adherence to HeLa cells. Infect Immun. 1992;60(6):2541–3. Epub 1992/06/01. 1587620 10.1128/iai.60.6.2541-2543.1992 PMC257194

[34] Eskelinen E-L. New Insights into the Mechanisms of Macroautophagy in Mammalian Cells. 2008;266:207–47. 10.1016/s1937-6448(07)66005-5 18544495

[35] Eskelinin. Fine structure of the autophagosome. Methods in Molecular Biology,. 2008b;445:11–28. 10.1007/978-1-59745-157-4_2 18425441

[36] Mizushima N, Yoshimori T, Levine B. Methods in mammalian autophagy research. Cell. 2010;140(3):313–26. Epub 2010/02/11. 10.1016/j.cell.2010.01.028 20144757 PMC2852113

[37] Knodler LA, Celli J. Eating the strangers within: host control of intracellular bacteria via xenophagy. Cell Microbiol. 2011;13(9):1319–27. Epub 2011/07/12. 10.1111/j.1462-5822.2011.01632.x 21740500 PMC3158265

[38] O'Seaghdha M, Wessels MR. Streptolysin O and its co-toxin NAD-glycohydrolase protect group A Streptococcus from Xenophagic killing. PLoS Pathog. 2013;9(6):e1003394 Epub 2013/06/14. 10.1371/journal.ppat.1003394 23762025 PMC3675196

[39] Weisner AM, Johnson AP, Lamagni TL, Arnold E, Warner M, Heath PT, et al Characterization of group B streptococci recovered from infants with invasive disease in England and Wales. Clin Infect Dis. 2004;38(9):1203–8. Epub 2004/05/06. 10.1086/382881 . 15127328

[40] Sheen TR, Jimenez A, Wang NY, Banerjee A, van Sorge NM, Doran KS. Serine-rich repeat proteins and pili promote Streptococcus agalactiae colonization of the vaginal tract. J Bacteriol. 2011;193(24):6834–42. Epub 2011/10/11. 10.1128/JB.00094-11 21984789 PMC3232834

[41] Doran K. Group B Streptococcal b-Hemolysin/Cytolysin Promotes Invasion of Human Lung Epithelial Cells and the Release of Interleukin-8. Journal of Infectious Diseases. 2002;185:196–203. 11807693 10.1086/338475

[42] Nagao. Group B Streptococcus induces tyrosine phosphorylation of annexin V and glutathione S-transferase in human umbilical vein endothelial cells. International Journal of Molecular Medicine. 2009;24(3). 10.3892/ijmm_00000245 19639233

[43] Rubens. Pathophysiology and Histopathology of Group B Streptococcal Sepsis in Macaca nemestrina Primates Induced after Intraamniotic Inoculation: Evidence for Bacterial Cellular Invasion. The Journal of Infectious Diseases. 1991;164(2):320–30. 1856481 10.1093/infdis/164.2.320

[44] Gutekunst H, Eikmanns BJ, Reinscheid DJ. The novel fibrinogen-binding protein FbsB promotes Streptococcus agalactiae invasion into epithelial cells. Infect Immun. 2004;72(6):3495–504. Epub 2004/05/25. 10.1128/IAI.72.6.3495-3504.2004 15155657 PMC415667

[45] Seo HS, Minasov G, Seepersaud R, Doran KS, Dubrovska I, Shuvalova L, et al Characterization of Fibrinogen Binding by Glycoproteins Srr1 and Srr2 of Streptococcus agalactiae. J Biol Chem. 2013;288(50):35982–96. Epub 2013/10/30. 10.1074/jbc.M113.513358 24165132 PMC3861647

[46] Mandlik A, Swierczynski A, Das A, Ton-That H. Pili in Gram-positive bacteria: assembly, involvement in colonization and biofilm development. Trends Microbiol. 2008;16(1):33–40. Epub 2007/12/18. 10.1016/j.tim.2007.10.010 18083568 PMC2841691

[47] Kline KA, Dodson KW, Caparon MG, Hultgren SJ. A tale of two pili: assembly and function of pili in bacteria. Trends Microbiol. 2010;18(5):224–32. Epub 2010/04/10. 10.1016/j.tim.2010.03.002 20378353 PMC3674877

[48] Claverys. A Type IV Pilus Mediates DNA Binding during Natural Transformation in Streptococcus pneumoniae. PLOS Pathogens. 2013;9(6):1–11. 10.1371/journal.ppat.1003473 PMC3694846 23825953

[49] Laur. Genome Analysis Reveals Pili in Group B Streptococcus. Science. 2005;309(5731):105 15994549 10.1126/science.1111563

[50] Barocchi MA, Ries J, Zogaj X, Hemsley C, Albiger B, Kanth A, et al A pneumococcal pilus influences virulence and host inflammatory responses. Proc Natl Acad Sci U S A. 2006;103(8):2857–62. Epub 2006/02/17. 10.1073/pnas.0511017103 16481624 PMC1368962

[51] Mora M, Bensi G, Capo S, Falugi F, Zingaretti C, Manetti AG, et al Group A Streptococcus produce pilus-like structures containing protective antigens and Lancefield T antigens. Proc Natl Acad Sci U S A. 2005;102(43):15641–6. Epub 2005/10/15. 10.1073/pnas.0507808102 16223875 PMC1253647

[52] Nobbs AH, Rosini R, Rinaudo CD, Maione D, Grandi G, Telford JL. Sortase A utilizes an ancillary protein anchor for efficient cell wall anchoring of pili in Streptococcus agalactiae. Infect Immun. 2008;76(8):3550–60. Epub 2008/06/11. 10.1128/IAI.01613-07 18541657 PMC2493207

[53] Reinert LS, Harder L, Holm CK, Iversen MB, Horan KA, Dagnaes-Hansen F, et al TLR3 deficiency renders astrocytes permissive to herpes simplex virus infection and facilitates establishment of CNS infection in mice. J Clin Invest. 2012;122(4):1368–76. Epub 2012/03/20. 10.1172/JCI60893 22426207 PMC3314467

[54] Yamamoto M, Kamatsuka Y, Ohishi A, Nishida K, Nagasawa K. P2X7 receptors regulate engulfing activity of non-stimulated resting astrocytes. Biochem Biophys Res Commun. 2013;439(1):90–5. Epub 2013/08/21. 10.1016/j.bbrc.2013.08.022 . 23958305

[55] Chung WS, Clarke LE, Wang GX, Stafford BK, Sher A, Chakraborty C, et al Astrocytes mediate synapse elimination through MEGF10 and MERTK pathways. Nature. 2013;504(7480):394–400. Epub 2013/11/26. 10.1038/nature12776 . 24270812 PMC3969024

[56] Esen N, Tanga FY, DeLeo JA, Kielian T. Toll-like receptor 2 (TLR2) mediates astrocyte activation in response to the Gram-positive bacterium Staphylococcus aureus. Journal of Neurochemistry. 2003;88(3):746–58. 10.1046/j.1471-4159.2003.02202.x 14720224

[57] Carpentier PA, Begolka WS, Olson JK, Elhofy A, Karpus WJ, Miller SD. Differential activation of astrocytes by innate and adaptive immune stimuli. Glia. 2005;49(3):360–74. Epub 2004/11/13. 10.1002/glia.20117 . 15538753

[58] Pinto A, Jacobsen M, Geoghegan PA, Cangelosi A, Cejudo ML, Tironi-Farinati C, et al Dexamethasone Rescues Neurovascular Unit Integrity from Cell Damage Caused by Systemic Administration of Shiga Toxin 2 and Lipopolysaccharide in Mice Motor Cortex. PLoS ONE. 2013;8(7):e70020 10.1371/journal.pone.0070020 23894578 PMC3720947

[59] Brega S, Caliot E, Trieu-Cuot P, Dramsi S. SecA localization and SecA-dependent secretion occurs at new division septa in group B Streptococcus. PLoS One. 2013;8(6):e65832 Epub 2013/06/14. 10.1371/journal.pone.0065832 23762438 PMC3676364

[60] Reinscheid DJ, Gottschalk B, Schubert A, Eikmanns BJ, Chhatwal GS. Identification and Molecular Analysis of PcsB, a Protein Required for Cell Wall Separation of Group B Streptococcus. Journal of Bacteriology. 2001;183(4):1175–83. 10.1128/jb.183.4.1175-1183.2001 11157929 PMC94990

[61] Stella. Interleukin-1 Enhances the ATP-Evoked Release of Arachidonic Acid from Mouse Astrocytes. The Journal of Neuroscience. 1997;17(9):2939–46. 9096130 10.1523/JNEUROSCI.17-09-02939.1997 PMC6573655

[62] Tanaka. Astrocytes prevent neuronal death induced by reactive oxygen and nitrogen species. GLIA. 1999;28(2):85–986. 10533053 10.1002/(sici)1098-1136(199911)28:2<85::aid-glia1>3.0.co;2-y

[63] Dinarello. Proinflammatory Cytokines. Chest. 2000;118(2):503–8. 10936147 10.1378/chest.118.2.503

[64] van Furth AM, Roord JJ, van Furth R. Roles of proinflammatory and anti-inflammatory cytokines in pathophysiology of bacterial meningitis and effect of adjunctive therapy. Infection and Immunity. 1996;64(12):4883–90. 8945522 10.1128/iai.64.12.4883-4890.1996 PMC174464

[65] Dudley. Inflammatory cytokine mRNA in human gestational tissues: implications for term and preterm labor. J Soc Gynecol Investig. 1996;Nov-Dec 3(6):328–35. 8923417

[66] Hunolstein v. Soluble antigens from group B streptococci induce cytokine production in human blood cultures. Infection and Immunity. 1997;65(10):4017–21. 9317001 10.1128/iai.65.10.4017-4021.1997 PMC175577

[67] Reisenberger. In vitro cytokine and prostaglandin production by amnion cells in the presence of bacteria. American Journal of Obstetrics & Gynecology. 1997;176(5):981–4. 9166155 10.1016/s0002-9378(97)70389-2

[68] Winram. Characterization of Group B Streptococcal Invasion of Human Chorion and Amnion Epithelial Cells In Vitro. Infection and Immunity. 1998;66(10):4932–41. 9746599 10.1128/iai.66.10.4932-4941.1998 PMC108610

[69] Zaga V, Estrada-Gutierrez G, Beltran-Montoya J, Maida-Claros R, Lopez-Vancell R, Vadillo-Ortega F. Secretions of interleukin-1beta and tumor necrosis factor alpha by whole fetal membranes depend on initial interactions of amnion or choriodecidua with lipopolysaccharides or group B streptococci. Biol Reprod. 2004;71(4):1296–302. Epub 2004/06/18. 10.1095/biolreprod.104.028621 . 15201199

[70] Dammann O, Leviton A. Maternal intrauterine infection, cytokines, and brain damage in the preterm newborn. Pediatric Research. 1997;42:1–8. 9212029 10.1203/00006450-199707000-00001

[71] Argaw AT. Astrocyte-derived VEGF-A drives blood-brain barrier disruption in CNS inflammatory disease. The Journal of Clinical Investigation 2012;122(7):2454–68. 10.1172/JCI60842 22653056 PMC3386814

[72] Zeng J. Induction of cytopathic effect and cytokines in coxsackievirus B3-infected murine astrocytes. Virology Journa;. 2013;10(157):1–8. 10.1186/1743-422X-10-157 23693026 PMC3680086

[73] Baggiolini M, Dewald B, Moser B. Interleukin-8 and related chemotactic cytokines—CXC and CC chemokines. Adv Immunol. 1994;55:97–179. Epub 1994/01/01. . 8304236

[74] Bian Y, Zhao X, Li M, Zeng S, Zhao B. Various roles of astrocytes during recovery from repeated exposure to different doses of lipopolysaccharide. Behav Brain Res. 2013;253:253–61. Epub 2013/07/31. 10.1016/j.bbr.2013.07.028 . 23896049

[75] Netea MG, Simon A, van de Veerdonk F, Kullberg B-J, Van der Meer JWM, Joosten LAB. IL-1β Processing in Host Defense: Beyond the Inflammasomes. PLoS Pathog. 2010;6(2):e1000661 10.1371/journal.ppat.1000661 20195505 PMC2829053

[76] Kaya D, Gursoy-Ozdemir Y, Yemisci M, Tuncer N, Aktan S, Dalkara T. VEGF protects brain against focal ischemia without increasing blood—brain permeability when administered intracerebroventricularly. J Cereb Blood Flow Metab. 2005;25(9):1111–8. Epub 2005/04/15. 10.1038/sj.jcbfm.9600109 . 15829918

[77] Martin TR, Rubens CE, Wilson CB. Lung Antibacterial Defense Mechanisms in Infant and Adult Rats: Implications for the Pathogenesis of Group B Streptococcal Infections in the Neonatal Lung. Journal of Infectious Diseases. 1988;157(1):91–100. 10.1093/infdis/157.1.91 3275727

[78] Pezzicoli A, Santi I, Lauer P, Rosini R, Rinaudo D, Grandi G, et al Pilus Backbone Contributes to Group B Streptococcus Paracellular Translocation through Epithelial Cells. Journal of Infectious Diseases. 2008;198(6):890–8. 10.1086/591182 18694342

[79] Wilkinson H. NontypableGroupB StreptococciIsolatedfromHuman Sources. Journal of Clinical Microbiology. 1977;6(2):183–4. 408376 10.1128/jcm.6.2.183-184.1977 PMC274732

[80] Madoff LC, Michel JL, Kasper DL. A monoclonal antibody identifies a protective C-protein alpha-antigen epitope in group B streptococci. Infection and Immunity. 1991;59(1):204–10. PubMed PMID: PMC257727. 1702759 10.1128/iai.59.1.204-210.1991 PMC257727

[81] Wessels MR, Paoletti LC, Rodewald AK, Michon F, DiFabio J, Jennings HJ, et al Stimulation of protective antibodies against type Ia and Ib group B streptococci by a type Ia polysaccharide-tetanus toxoid conjugate vaccine. Infection and Immunity. 1993;61(11):4760–6. PubMed PMID: PMC281231. 8406875 10.1128/iai.61.11.4760-4766.1993 PMC281231

[82] Jiang SM, Ishmael N, Dunning Hotopp J, Puliti M, Tissi L, Kumar N, et al Variation in the group B Streptococcus CsrRS regulon and effects on pathogenicity. J Bacteriol. 2008;190(6):1956–65. Epub 2008/01/22. 10.1128/JB.01677-07 18203834 PMC2258897

[83] Bensing BA, Gibson BW, Sullam PM. The Streptococcus gordonii Platelet Binding Protein GspB Undergoes Glycosylation Independently of Export. Journal of Bacteriology. 2004;186(3):638–45. 10.1128/jb.186.3.638-645.2004 14729688 PMC321503

[84] Llull D, Garcia E, Lopez R. Tts, a processive beta-glucosyltransferase of Streptococcus pneumoniae, directs the synthesis of the branched type 37 capsular polysaccharide in Pneumococcus and other gram-positive species. J Biol Chem. 2001;276(24):21053–61. Epub 2001/03/27. 10.1074/jbc.M010287200 . 11264282

